# Expression of Gal-9 on Dendritic Cells and Soluble Forms of TIM-3/Gal-9 in Patients Suffering from Endometriosis

**DOI:** 10.3390/ijms24065948

**Published:** 2023-03-21

**Authors:** Dorota Suszczyk, Wiktoria Skiba, Anna Pawłowska, Grzegorz Polak, Rafał Tarkowski, Iwona Wertel

**Affiliations:** 1 Independent Laboratory of Cancer Diagnostics and Immunology, Medical University of Lublin, Chodźki 1, 20-093 Lublin, Poland; 2 I Chair and Department of Gynaecologic Oncology and Gynaecology, Medical University of Lublin, Staszica 16, 20-081 Lublin, Poland

**Keywords:** endometriosis, dendritic cells, galectin-9, immunosuppression, peritoneal cavity, inflammation

## Abstract

Immune system dysregulation is clinically evident in the pathogenesis of endometriosis (EMS). Changes in the dendritic cells (DCs) activity or phenotype may be involved in the implantation and growth of endometrial tissue outside the uterus in the disease. The TIM-3/Gal-9 axis is implicated in the development of immune tolerance. However, the knowledge about the exact role of this pathway in the EMS is extremely poor. In the present study, we evaluated the expression of Gal-9 on myeloid DCs (mDCs) and plasmacytoid DCs (pDCs) in the peripheral blood (PB) and peritoneal fluid (PF) of both EMS patients (*n* = 82) and healthy subjects (*n* = 10) via flow cytometry. We also investigated the concentrations of soluble Gal-9 and TIM-3 in the plasma and PF of EMS patients and the control group using ELISA. We showed significantly elevated percentages of mDCs-Gal-9^+^ and pDCs-Gal-9^+^, and significantly higher concentrations of the soluble form of Gal-9 and TIM-3 in the PF of EMS patients than in circulation. Our results led us to conclude that the accumulation of Gal-9 expressing mDCs and pDCs in the PF and high sTIM-3/Gal-9 production in the peritoneal cavity could represent the hallmark of immune regulation in EMS patients, which may augment the inflammatory process and development/maintenance of local immunosuppression.

## 1. Introduction

Endometriosis (EMS) is a chronic inflammatory disorder characterized by the growth of endometrial cells ectopically outside the uterus as “endometrial-like lesions”. It is associated with chronic pelvic pain, described as severe and progressive; dyspareunia; dysmenorrhea; gastrointestinal disorders; and infertility [1]. EMS affects 10% of women of reproductive age, 50% of women with infertility, and 50–80% of women with chronic pelvic pain [2]. Although EMS is still called the “disease of a thousand hypotheses”, and many theories seek to explain the emergence and development of this disease, none of them clearly explain the survival and proliferation mechanism of the ectopic endometrium implants beyond their natural place.

The development of EMS is accompanied by multifactorial changes in the systemic and local immunity, as well as by disturbances of the proper activity of immune cells, such as monocytes/macrophages (Mo/Ma), neutrophils, B cells, T cells, natural killer (NK) cells, myeloid-derived suppressor cells (MDSCs), and dendritic cells (DCs) [3,4,5].

DCs provide a link between the innate and adaptive immune response. They play a predominant role in the antitumor immune response via both the priming and activation of T cells. During cancer progression, DCs are able to switch their immunostimulatory activity into the immunosuppressive. However, the mechanism of the transition is still unknown [6,7]. The disturbances in the quantity, maturity, and activity of DCs subsets may be involved in the implantation and growth of endometrial tissue outside the uterus by the non-efficient clearing of endometrial cells, the induction of tolerance, and/or immunosupression [8].

A growing body of evidence focuses on the implication of immune checkpoints (ICPs) such as PD-1/PD-L1/PD-L2 in cancer development and also in EMS [8,9,10,11,12]. ICPs are crucial in controlling numerous biological processes, as well as maintaining the balance between the immune response and the tolerance of self-tissue in healthy conditions. Co-inhibitory receptors may promote anergy in T lymphocytes, hamper T cell activation, and have an impact on the ability to prime T cells by antigen-presenting cells (APCs) or the secretion of various cytokines by T cells [13]. In our previous paper, we evaluated the role of the PD-1/PD-L1/PD-L2 pathway in the pathogenesis of EMS [8]. Despite significant advances in the field of the immunological aspects of endometriosis, little is known about the role of the T cell immunoglobulin and mucin domain-containing protein/galectin-9 (TIM-3/Gal-9) pathway in this disease.

Gal-9 is a ligand for TIM-3 and can be found on the surface of eosinophils, T cells, Ma, or DCs [14]. It regulates the maturation of DCs from immature DCs (iDCs) and the differentiation of regulatory T cells (Tregs) or Th17 cells [15]. Moreover, Gal-9 increases the infiltration of immunosuppressive cells, such as MDSCs, Ma, and plasmacytoid DC-like macrophages, playing a pivotal role in the development of immunosuppression. In the tumor microenvironment, the interaction between the Gal-9 and TIM-3 present on DCs inhibits antitumor immunity [16]. Gal-9 negatively regulates the Th1 responses, leading to the induction of T cell apoptosis and exhaustion [15,17]. Interestingly, the higher concentration of Gal-9 may lead to the apoptosis of the activated CD4^+^ and CD8^+^ T cells, whereas the lower concentration may have a stimulatory effect, increasing the production of cytokines by the activated T cells. In addition, Gal-9 is concerned with the development of autoimmune diseases [18]. In DCs, the TIM-3/Gal-9 pathway synergizes with Toll Like Receptors (TLRs) to promote inflammation. It was proven that chronic inflammation is the hallmark feature of EMS [19,20]. Blocking TIM-3 cramps the development of immunotolerance via Th1 cells and may be the key point in restoring the proper function of the exhausted T cells [21,22].

Recent evidence has shown that Gal-9 mRNA was overexpressed in the eutopic endometrium of EMS patients. Additionally, in ectopic implants, the Gal-9 mRNA was higher than in the cells surrounding the implantation area, suggesting that implants are the source of Gal-9 [23]. Disturbances in the TIM-3/Gal-9 pathway may lead to the loss of control under this process, consequently favoring the invasiveness of endometrial implants [24].

Based on the research outcomes regarding the role of TIM-3/Gal-9 in inflammatory or autoimmunological diseases and cancers, we hypothesize that the TIM-3/Gal-9 pathway may play a significant role in the implantation and growth of endometrial-like lesions and the progression of endometriosis.

The aim of our research was to evaluate the expression of galectin-9 on myeloid (mDCs) and plasmacytoid (pDCs) dendritic cells and their distribution in both the systemic (peripheral blood, PB) and local peritoneal environment (peritoneal fluid, PF) of patients with EMS, in relation to the American Society for Reproductive Medicine (ASRM) stages, and healthy women. We also evaluated the concentration of the soluble form of Gal-9 and TIM-3 in the plasma and PF of EMS patients and the plasma of healthy blood donors in terms of their clinical value.

## 2. Results

### 2.1. Distribution of Myeloid and Plasmacytoid Dendritic Cells with Gal-9 Expression in Patients with Endometriosis

**Figure 1:**
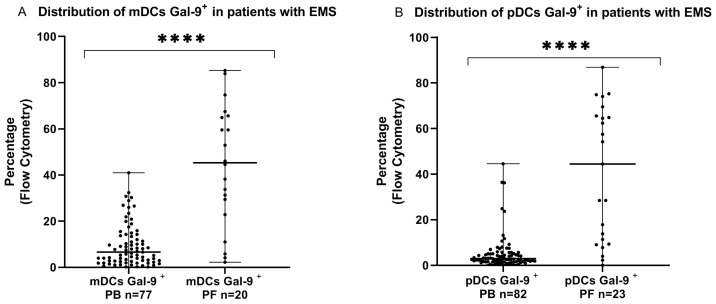
Percentage of mDCs (**A**) and pDCs (**B**) with Gal-9 expression in peripheral blood and peritoneal fluid in patients suffering from endometriosis. The median values with the following signs indicate statistically significant differences: **** *p* < 0.0001.

Firstly, we conducted the examination of the dendritic cells with Galectin-9 expression in the systemic and local peritoneal environment of patients with endometriosis. We showed that the percentage of myeloid and plasmacytoid DCs Gal-9^+^ was significantly higher in the peritoneal fluid than in the peripheral blood (*p* < 0.0001) (Figure 1).

### 2.2. Percentage of Myeloid and Plasmacytoid Dendritic Cells with Gal-9 Expression in Relation to ASRM Stages of Endometriosis

Secondly, we examined the percentage of mDCs Gal-9^+^ and pDCs Gal-9^+^ subpopulations in PB and PF in the early (I/II), and late (II/IV) ASRM stages of endometriosis. We did not observe statistically significant differences (*p* > 0.05) in the percentages of the mDCs Gal-9^+^ and pDCs Gal-9^+^ subpopulation between the early (I/II) and late (III/IV) ASRM stages of EMS in both the peripheral blood and peritoneal fluid (Figure S1).

### 2.3. Percentage of Myeloid and Plasmacytoid Dendritic Cells with Gal-9 Expression in Patients with Endometriosis and the Control Group

**Figure 2:**
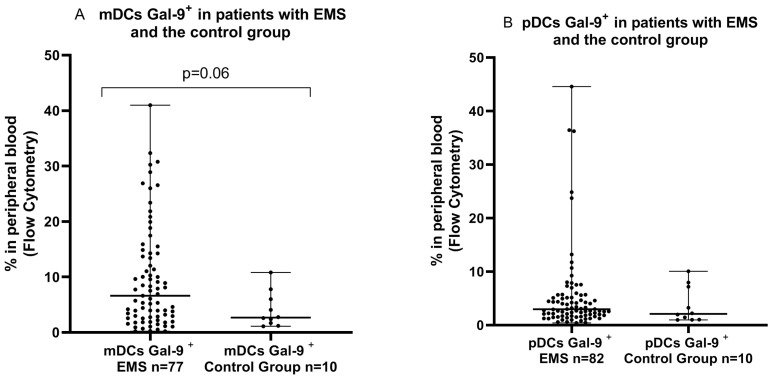
Percentage of mDCs Gal-9-positive (**A**) and pDCs Gal-9-positive cells (**B**) in peripheral blood in patients with endometriosis and control group.

The percentage of mDCs Gal-9^+^ was higher in the EMS group than in the PB of the healthy subjects; however, the difference was near the statistically significant point (*p* = 0.06593). There was no significant difference (*p* > 0.05) in the percentages of Gal-9-positive pDCs in the PB of patients with EMS in comparison to the PB of the control group (Figure 2).

### 2.4. Percentage of Myeloid and Plasmacytoid Dendritic Cells with Gal-9 Expression in Relation to ASRM Stages of Endometriosis and the Control Group

**Figure 3:**
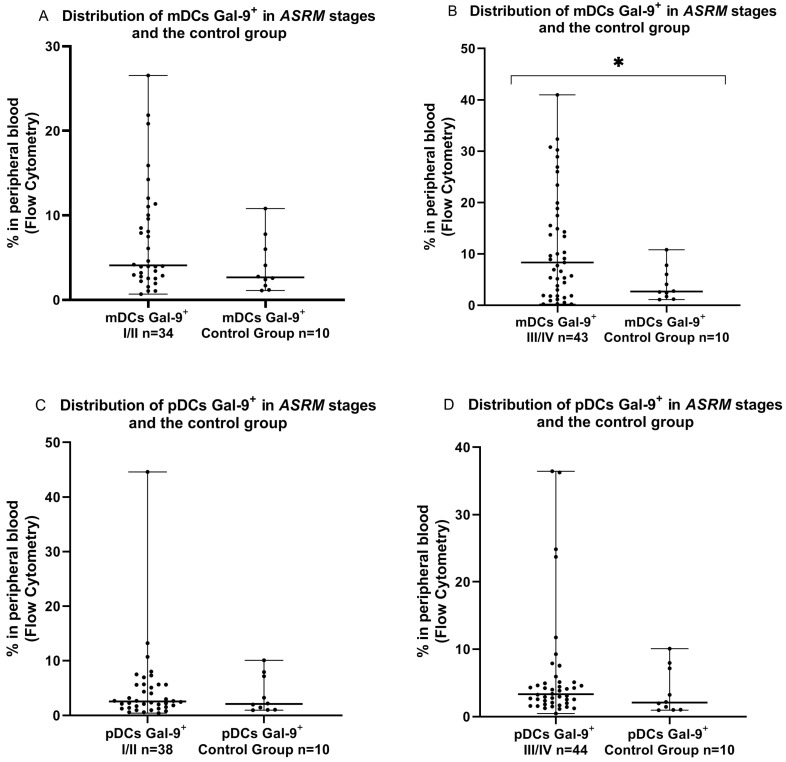
Percentage of mDCs Gal-9-positive and pDCs Gal-9-positive cells in peripheral blood (**A**–**D**) in early (I/II) and late (III/IV) ASRM stages of EMS and in the control group. The median values with the following sign indicate statistically significant differences: * *p* < 0.05.

Next, we performed an examination of the mDCs Gal-9-positive and pDCs Gal-9-positive cells in the PB in the early (I/II) and late (II/IV) ASRM stages of endometriosis in comparison to the PB from healthy women. Interestingly, we demonstrated that mDCs Gal-9^+^ was significantly higher (*p* < 0.05) in the late (III/IV) ASRM stages of EMS than that determined in the PB of the control group. We did not observe a statistically significant difference (*p* > 0.05) in the percentages of Gal-9-positive mDCs in the early (I/II) and Gal-9-positive pDCs in both the early (I/II) and late (III/IV) ASRM stages in the PB of patients with EMS in comparison to the PB of the control group (Figure 3).

The percentage summary of Gal-9 expression on the mDCs and pDCs of the EMS patients and the control group can be found in the table below (Table 1).

### 2.5. Concentration of the Soluble Form of Gal-9 in Plasma and Peritoneal Fluid of Patients with Endometriosis

**Figure 4:**
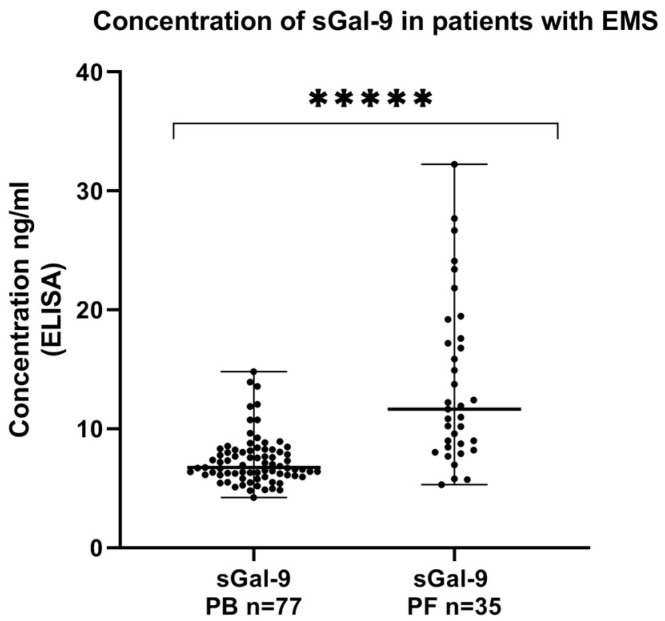
Levels of sGal-9 (ng/mL) in the plasma and peritoneal fluid of patients with endometriosis. The median value with the following signs indicate statistically significant differences: ***** *p* < 0.00001.

We showed that the concentration of sGal-9 in the peritoneal fluid was significantly higher than in the plasma of the EMS patients (*p* < 0.00001) (Figure 4).

### 2.6. Concentration of sGal-9 in the Plasma and Peritoneal Fluid in Relation to ASRM Stages of Endometriosis

In the next step, we compared the concentrations of sGal-9 in the peripheral blood and peritoneal fluid in the early (I/II) and late (III/IV) ASRM stages of endometriosis. The concentration of sGal-9 in the plasma and peritoneal fluid did not differ significantly between the early (I/II) and late (III/IV) ASRM stages of endometriosis (*p* > 0.05) (Figure S2).

### 2.7. Concentration of sGal-9 in Plasma of Patients with Endometriosis and the Control Group

**Figure 5:**
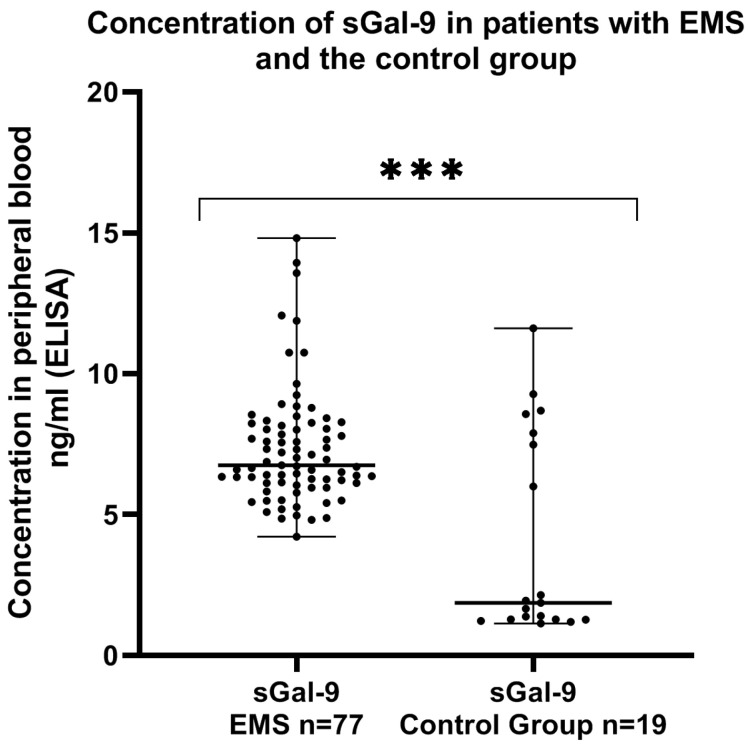
Levels of sGal-9 (ng/mL) in the plasma of patients with endometriosis and control group. The median value with the following signs indicate statistically significant differences: *** *p* < 0.001.

Next, we compared the concentration of sGal-9 in the plasma of EMS patients and the control group. The concentration of sGal-9 was significantly higher (*p* < 0.001) in the plasma of the EMS patients than that detected in the control group (Figure 5).

### 2.8. Concentration of sGal-9 in Plasma of Patients with Early (I/II) and Late (III/IV) ASRM Stages of EMS and the Control Group

**Figure 6:**
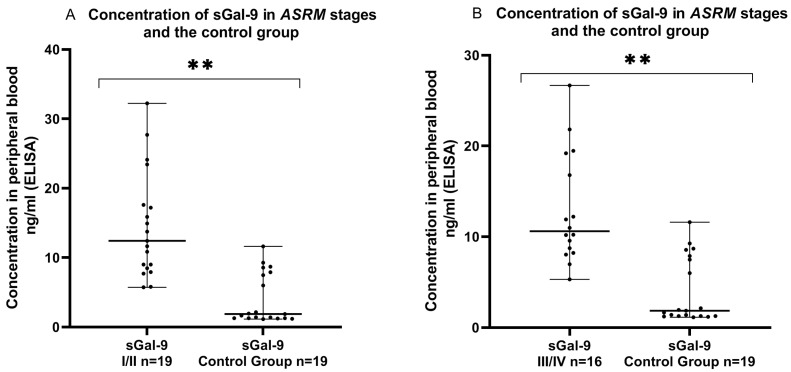
Level of sGal-9 in the plasma in early (I/II) (**A**) and late (III/IV) (**B**) ASRM stages of endometriosis and in the plasma of the control group (ng/mL). The median value with the following sign indicate statistically significant differences: ** *p* < 0.01.

Finally, we examined the concentration of sGal-9 in the plasma in the early (I/II) and late (III/IV) ASRM stages of EMS patients and in the control group. The concentration of plasma sGal-9 in the early (I/II) ASRM stages of EMS was significantly higher (*p* < 0.01) than that detected in the plasma of the control group. Similarly, the concentration of sGal-9 was also significantly elevated (*p* < 0.01) in the plasma in the late (III/IV) ASRM stages of endometriosis in comparison to the control group (Figure 6).

### 2.9. Concentration of the Soluble Form of TIM-3 in Plasma and Peritoneal Fluid of Patients with Endometriosis

**Figure 7:**
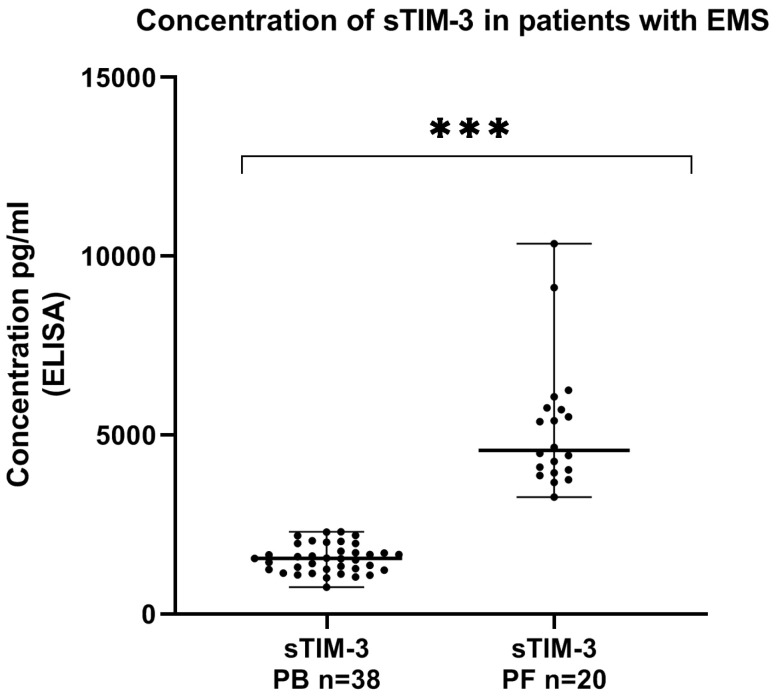
Levels of sTIM-3 (pg/mL) in the plasma and peritoneal fluid of patients with endometriosis. The median value with the following sign indicates statistically significant difference: *** *p* < 0.001.

We showed that the concentration of sTIM-3 in the peritoneal fluid was significantly higher than in the plasma of EMS patients (*p* < 0.001) (Figure 7).

### 2.10. Concentration of sTIM-3 in the Plasma and Peritoneal Fluid in Relation to ASRM Stages of Endometriosis

In the further step, we compared the concentrations of sTIM-3 in the peripheral blood and peritoneal fluid in the early (I/II) and late (II/IV) ASRM stages of endometriosis. The concentration of sTIM-3 in the plasma and peritoneal fluid did not differ significantly between the early (I/II) and late (III/IV) ASRM stages of endometriosis (*p* > 0.05) (Figure S3).

### 2.11. Concentration of sTIM-3 in Plasma of Patients with Endometriosis and the Control Group

**Figure 8:**
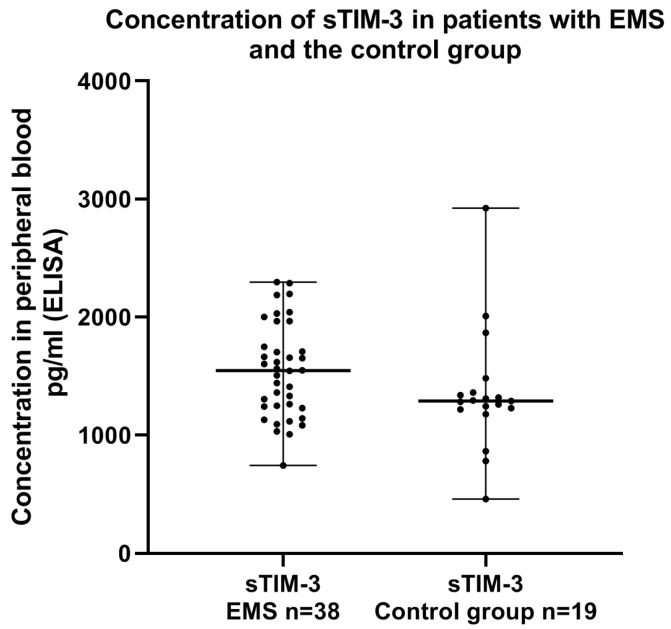
Levels of sTIM-3 (pg/mL) in the plasma of patients with endometriosis and the control group.

Next, we compared the concentration of sTIM-3 in the plasma of EMS patients and the control group. The concentration of sTIM-3 was higher in the plasma of the EMS patients than in the plasma of the control group; however, the difference was not statistically significant (*p* > 0.05) (Figure 8).

### 2.12. Concentration of sTIM-3 in Plasma of Patients with Early (I/II) and Late (III/IV) ASRM Stages of EMS and the Control Group

**Figure 9:**
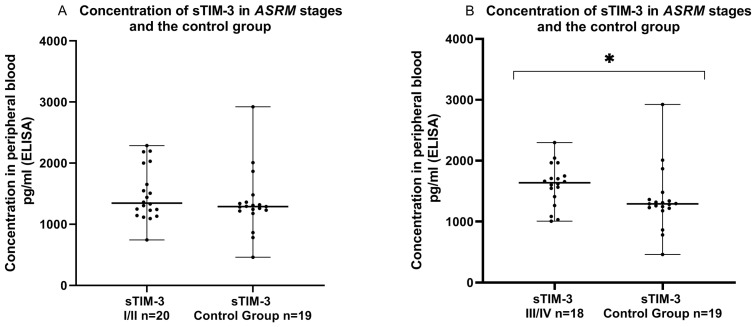
Level of sTIM-3 in the plasma in early (I/II) (**A**) and late (III/IV) (**B**) ASRM stages of endometriosis and in the plasma of the control group (pg/mL). The median value with the following sign indicate statistically significant difference: * *p* < 0.05.

Finally, we examined the concentration of sTIM-3 in the plasma in the early (I/II) and late (III/IV) ASRM stages of EMS patients and in the control group. We showed that the level of sTIM-3 was higher in the plasma in the late stages (III/IV) of EMS than that detected in the plasma of the control group, and reached the significance level (*p* > 0.05). Similarly, the concentration of sTIM-3 in the plasma was higher in the early (I/II) ASRM stages of EMS patients in comparison to the healthy subjects; however, the difference was not statistically significant (*p* > 0.05) (Figure 9).

The summary of the sGal-9 and TIM-3 concentrations of the EMS patients and the control group can be found in Table 2.

## 3. Discussion

Endometriosis is a multifactorial heterogeneous disease. However, the role of inflammation and the potential immune system dysregulation in its pathogenesis is clinically evident. Women with EMS are noted to have altered peripheral and peritoneal cells activity, including the reduced cytotoxicity of the NK cells or decreased T cell reactivity, which lead to the failure in removing fragments of the endometrium from the peritoneal cavity and induces excessive local inflammation [25]. In our previous research study, we observed a higher frequency of mDCs and pDCs with PD-L1 and PD-L2 expression in the PF of patients with EMS [8]. In the current study, we explored the expression of Gal-9 on the mDCs and pDCs in both the systemic and local peritoneal environment of EMS patients. The obtained data were referred to the clinical characteristics of patients and compared with the data from healthy women to assess their clinical value.

We showed that the levels of mDCs and pDCs with Gal-9 expression were elevated in the PF, in comparison to the PB, of EMS patients. Nevertheless, we did not observe statistically significant differences in the percentages of mDCs Gal-9^+^ nor pDCs Gal-9^+^ subpopulations between the early (I/II) and late (III/IV) ASRM stages of EMS, in both the PB and PF. To the best of our knowledge, this is the first study examining the expression of this lectin on both DC subsets, in two different environments, in patients suffering from EMS.

Meggyes et al., using flow cytometry, showed an altered distribution of lymphocytes in women with and without EMS. They established the elevated expression of Gal-9 on CD4^+^ T cells and Tregs in the PB of EMS patients in comparison to healthy subjects. Additionally, the authors demonstrated a higher expression of Gal-9 on the peritoneal CD8^+^ T cells and NK cells compared with the PB [26]. Moreover, peritoneal T cell subsets showed an increased TIM-3 expression than in the PB. The authors also revealed elevated TIM-3 expression on peritoneal NKT-like cells in the EMS group [26]. Confronting the literature data with our findings, we suggest that the accumulation of Gal-9 expressing mDCs and pDCs in the peritoneal cavity could represent the hallmark of the immune regulation in women with endometriosis.

Interestingly, the available evidence suggests that the reactivity of the immune system is higher in women than in men [27]. DCs are strong promoters of self-tolerance via the production of tolerogenic cytokines or controlling the differentiation of Tregs, and disturbances in their proper activity may lead to the “breaking of tolerance” and the development of autoimmune diseases [5]. Recent evidence has established that women with EMS have an increased risk of developing rheumatoid arthritis (RA), systemic lupus erythematosus (SLE), multiple sclerosis (MS), and inflammatory bowel disease (IBD), in comparison with the general population. The presence of EMS can also be related to a higher risk of the co-occurrence of celiac disease (CLD), Sjögren’s disease (SS), or Addison’s disease [28].

Brubel et al., showed that Gal-9 mRNA was overexpressed in the eutopic endometrium of the EMS patients compared with the control group. The authors showed an elevated Gal-9 expression in the cells isolated from the PF of EMS patients. These results suggest that not only the eutopic endometrium or ectopic implants, but also peritoneal immune cells, may be the source of Gal-9 in patients with EMS. [23].

In our study, we also examined the soluble form of Gal-9 and TIM-3. Interestingly, we showed greater elevated levels of sGal-9 in the PF than in the plasma of EMS patients. The concentration of sGal-9 in the PF was nearly 3.6× times higher in comparison to that in the plasma of the EMS patients. Our results did not demonstrate relationships between the Gal-9 concentration in the early (I/II) and late (III/IV) ASRM stages of EMS in both the plasma and PF. Based on the received results, the level of sGal-9 could not be a useful marker of the disease progression.

However, it is worth highlighting the significantly higher concentration of sGal-9 in the plasma of the EMS patients in comparison to the healthy subjects. Additionally, we demonstrated an elevated level of plasma sGal-9 in the early (I/II) and late (III/IV) ASRM stages of EMS than in the control group.

Similar to our results, Brubel et al., showed an elevated level of sGal-9 in the serum of EMS patients in comparison to healthy women. They did not observe a relationship between the level of sGal-9 and EMS symptoms, such as chronic pelvic pain, dyspareunia, dysmenorrhea, dyschezia, and dysuria [23]. In their study, the concentration of serum sGal-9 was elevated only in the late (III/IV) ASRM stages of EMS compared with the control group. Contrary to Burbel et al., we observed significant differences in the plasma sGal-9 level between both the early (I/II) and the late (III/IV) ASRM stages of EMS and the control group [23]. It seems possible that Gal-9 in the early stages of EMS could trigger local inflammation and the migration of neutrophils, eosinophils, NK cells, T cells, Ma, and DCs into the peritoneal cavity. An elevated level of Gal-9 may be associated with immune imbalance, Th2 immune response dominance, and the progression of this disease [23]. Therefore, further research is necessary to describe the Gal-9 mechanism of action in EMS.

Jarollahi et al. (2022), in a case-control study, presented that the serum level of sGal-9 had high sensitivity (100%) and sufficient specificity (88.46%) to be a useful marker in the diagnostics of EMS compared to laparoscopy [29]. On the other hand, Brubel et al., detected the elevated concentration of sGal-9 in the serum, not only in EMS patients, but also in benign gynecologic conditions like infertility or chronic pelvic pain. In these cases, the use of Gal-9 as the diagnostic marker is insufficient to diagnose or confirm EMS [23].

The soluble form of Gal-9 was widely examined in autoimmune diseases and focused on clinical implications. Gal-9 is highly expressed in the synovial fluid and tissue of patients suffering from rheumatoid arthritis (RA) in comparison to healthy or osteoarthritic synovial tissue [30]. O’Brien et al., using a murine model of angiogenesis, demonstrated that the Gal-9 medium was responsible for inducing monocyte migration and acute inflammation after administration to mouse knees, highlighting the role of Gal-9 in the stimulation of angiogenesis and acute inflammation in arthritis [31]. Furthermore, the elevated Gal-9 level was demonstrated in patients with autoimmune hepatitis, primary Sjögren’s syndrome, and systemic and multiple sclerosis [32]. In patients with lupus erythematosus (SLE), the level of sGal-9 correlated with the systemic disease activity and might be considered as a discriminator between SLE patients with and without organ damage [33]. An elevated serum level of sGal-9 in systemic sclerosis (SSc) is associated with higher mortality, organ involvement, Th2-predominant immune imbalance, and the progression of this disease [34]. It should be underlined that EMS has some features of an autoimmune disease, manifested by tissue damage and the production of autoantibodies against endometrium, histones, ovary, and phospholipids, and may be associated with other autoimmune diseases [28,34,35,36].

An increased level of circulating sGal-9 has also been reported in cancers. An elevated concentration of sGal-9 was detected in the serum of patients with nasopharyngeal carcinoma in comparison with healthy subjects [37]. Reyes Vallejo et al., suggested that serum sGal-9 may participate in the progression of cervical cancer and may be a useful prognostic biomarker [38]. The accumulation of sGal-9 in the PF in our cohort of EMS patients highlights the important role of the soluble form of this lectin in the local peritoneal cavity environment.

In our study, we observed a greater accumulation of sTIM-3 in the PF than in the plasma of EMS patients. The concentration of sTIM-3 in the PF was nearly 3× times higher in comparison to the plasma of the EMS patients. However, its level did not differ significantly between the early (I/II) and late (III/IV) ASRM stages of EMS in both environments. Additionally, we reported significantly higher plasma sTIM-3 levels in the late (III/IV) ASRM stages of EMS than in the control group.

It is worth highlighting that the assessment of the soluble form of TIM-3 in the plasma and PF samples obtained from EMS patients was performed for the first time in our study. Even though the temporal expression of TIM-3 was found on activated T cells, the permanent expression of this receptor was observed after chronic stimulation. An increased TIM-3 expression leads to the suppression of the T cell response and “T cell exhaustion” [39]. Disturbances in TIM-3 expression have been described in autoimmune diseases. Li et al., showed the elevated expression of TIM-3 on CD4^+^ and CD8^+^ T cells in the PB and SF of patients suffering from RA, and its negative correlation with the severity of the disease [40]. Zhao et al., showed significantly increased levels of TIM-3 in the plasma of SLE patients and patients with active SLE, compared with the control group, suggesting that sTIM-3 may be involved in the disease progression and used as a possible biomarker to differentiate active/stable SLE [41].

**Figure 10:**
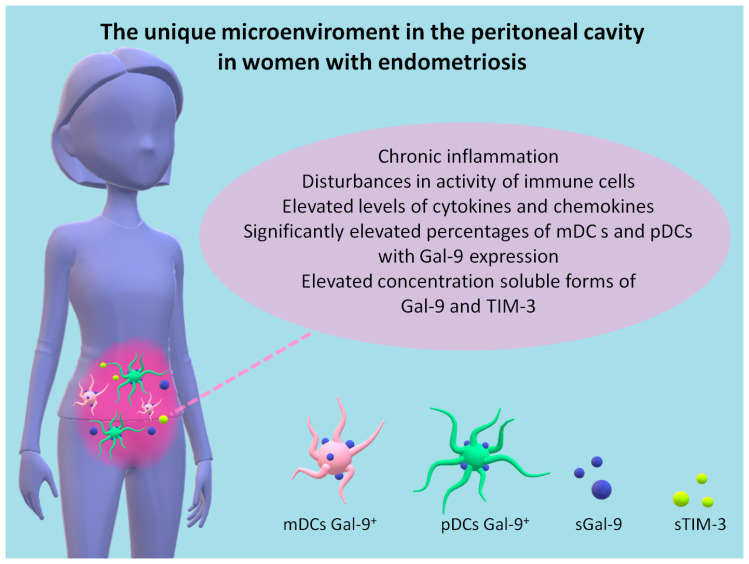
The unique microenvironment in the peritoneal cavity in women with endometriosis.

The key features of EMS include the presence of an increased PF volume with chronic peritoneal inflammation, disturbances of immune cells, secreted cytokines, and growth factors, which are critical for the survival of ectopic endometrial implants [42]. In our previous research study, we showed an accumulation of mDCs, mDCs, and pDCs with PD-L1 and PD-L2 expression, in the PF of EMS patients [8]. In the present study, we observed the accumulation of mDCs-Gal-9^+^, pDCs-Gal-9^+^, and a soluble form of Gal-9 and TIM-3 in the PF of EMS patients in comparison to the periphery (Figure 10).

These differences support the existence of immunological heterogeneity in endometriosis. The accumulation of sGal-9 and sTIM-3 observed in our study group in the PF may create a microenvironment that supports the implantation of endometrial tissue. The TIM-3/Gal-9 pathway may also be essential in the development of immunosuppression in EMS.

Understanding the microenvironment of the peritoneal cavity and the interactions between the lesions and immune cells in EMS patients will open the door to discovering new therapeutic methods based on immune modulators. It is known that EMS is associated with a higher risk of developing epithelial ovarian cancer (OC). Implants of the endometrium have shown characteristics similar to OC, i.e., the presence of neoangiogenesis, the invasion of tissues, chronic inflammation, uncontrolled growth, decreased apoptosis, and an elevated risk of recurrence [43,44,45]. We hope that non-hormonal treatment may help to break the immunosuppression in patients with EMS and redirect the immune system activity against endometrial implants.

## 4. Materials and Methods

### 4.1. Patients and the Control Group

The study group included 82 patients with endometriosis from the 1st Department of Oncological Gynaecology and Gynaecology Independent Public Clinical Hospital No. 1, Lublin, Poland. The diagnosis of EMS was confirmed by the histopathological reports and classified into one of four stages (I-minimal, II-mild, III-moderate, and IV-severe) according to the scale recommended by The American Society for Reproductive Medicine (ASRM). The criteria adopted for exclusion from the study included allergy and autoimmune diseases, active infections, pregnancy, and cancer. The control group consisted of 10 healthy subjects without gynecological diseases, allergy, autoimmune diseases, active infections, and pregnancy. Moreover, on the day of the experiment, the women had been regularly menstruating and did not declare symptoms of menopause. Patients were also chosen by matching age range, which was similar to the age range of the patients with EMS (Table 3). The control group was obtained thanks to the Regional Centre of Blood Donation and Blood Treatment in Lublin.

The study was approved by the Bioethics Committee of the Medical University of Lublin (KE-0254/116/2019, KE-0254/171/2021). The patients received the relevant information about the investigation and using biological material for scientific examinations and signed the written acquiescence form to participation.

### 4.2. Material

Peripheral blood samples from the EMS patients were collected into potassium EDTA S-Monovette tubes (Sarstedt, Nümbrecht, Germany) before the laparoscopy procedure. The PF was collected aseptically into sterile 15 mL Falcon (Wuxi NEST Biotechnology, Wuxi, China) during surgery.

The mononuclear cells (MNCs) from the PB and PF were separated immediately by density gradient centrifugation at 700× *g* with a Gradisol L (AquaMedica, Łódź, Poland) for 20 min at room temperature. Next, the interphase cells from the PB and PF were collected, washed twice, and resuspended in phosphate-buffered saline (PBS, PAA Laboratories GmbH, Pasching, Austria). MNCs from the healthy subjects were similarly isolated. A more detailed isolation protocol was described in our previous study [12].

The viability of the isolated cells was tested by using 470 µL of PBS, a 25 µL cells suspension, and a 5 µL Trypan Blue Solution (Sigma-Aldrich, St. Louis, MO, USA), counted in a Neubauer chamber. The MNCs were isolated within 2 h of collection and used for the flow cytometry analysis.

### 4.3. Flow Cytometry Analysis

To perform the flow cytometry analysis, the MNCs (1 × 10^6^ cells) were transferred into tubes and incubated with a combination of the following monoclonal antibodies, (mAbs) for 20 min at room temperature: anti-BDCA-1 FITC (anti-CD1c; clone: AD5-8E7, cat: 130-113-301; MACS MilenyiBiotec, Bergisch Gladbach, Germany), anti-BDCA-2 FITC (anti-CD303; clone: AC144, cat:130-113-192; MACS MilenyiBiotec), anti-CD19 PE-Cy5 (clone: SJ25C1, cat: 566396; BD Bioscience, Franklin Lakes, NJ, USA), anti-CD123 PE-Cy7 (clone: 6H6, cat: 306010; BioLegend, San Diego, CA, USA), anti-Gal-9 PE (clone:9M1-3, cat:348906; BioLegend, San Diego, CA, USA), anti-Gal-9 PE-Cy7 (clone:9M1-3, cat:348916; BioLegend, San Diego, CA, USA). After incubation, the cells were washed twice with PBS and analyzed as myeloid (BDCA-1^+^CD19^−^) and plasmacytoid (BDCA-2^+^CD123^+^) DCs with Gal-9 expression via flow cytometry (FACSCanto I Becton Dickinson, Franklin Lakes, NJ, USA) using FacsDiva software v6.1.3. During analysis 100,000 events were acquired.

**Figure 11:**
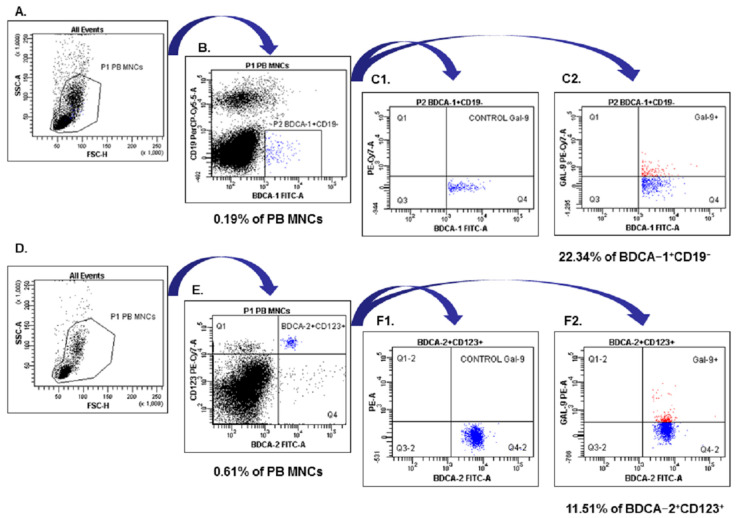
Flow cytometric analysis of BDCA-1^+^CD19^−^ and BDCA-2^+^CD123^+^ dendritic cells with Gal-9 expression in the PB of the patient with EMS. An acquisition gate was established based on FSC and SSC that included mononuclear cells (P1 population).

The P1 gated PB MNCs were analyzed for BDCA-1^+^CD19^−^ (region P2; (B)) and BDCA-2^+^CD123^+^ (region Q2; E). The final dot plots were drawn of BDCA-1^+^CD19^−^ Gal-9^+^ mDCs (region Q2; (C2)) and BDCA-2^+^CD123^+^Gal-9^+^ pDCs (region Q2-2, (F2)). Fluorescence minus one (FMO) control ((C1, F1), respectively) was used to verify the staining specificity and as a guide for setting the markers to delineate positive populations. The results were presented as the percentage of the total respective cell subsets i.e., myeloid BDCA-1^+^CD19^−^, plasmacytoid BDCA-2^+^CD123^+^ DCs with Gal-9 expression on the Figure 11.

### 4.4. ELISA

The concentration of the soluble forms of Gal-9 (cat: DGAL90; R&D Systems, Minneapolis, MN, USA; detection range: 0.2–10 ng/mL; sensitivity: 0.028 ng/mL) and TIM-3 (cat: DTIM30; R&D Systems, Minneapolis, MN, USA; detection range: 31.3–2000 pg/mL; sensitivity: 8.75 pg/mL) in the plasma and PF from the EMS patients and the plasma from the healthy donors were examined by the immunoassay kits following the manufacturer’s protocol. All samples were assayed in duplicate. The examination was performed on an ELX-800 plate reader (BioTek Instruments, Inc., Winooski, VT, USA) and analyzed using Gen5 (BioTek Instruments, Inc., Winooski, VT, USA).

### 4.5. Statistical Analysis

The obtained data were analyzed by Statistica 12.0 PL. The Wilcoxon paired test was used to compare the results from the PB and PF (the EMS group). The Mann–Whitney U test was chosen with respect to the results of the statistical comparison between the two studied groups (the EMS group/the control group). To obtain the relationships between the two parameters, Spearman’s rank correlation test was used. The data were presented as the median, minimum, and maximum values. A *p*-value of <0.05 was considered statistically significant.

## 5. Conclusions

In our study, we showed the differences in the distribution of myeloid and plasmacytoid DCs with Gal-9 expression in patients suffering from endometriosis. We established an accumulation of Gal-9 expressing mDCs and pDCs in the peritoneal cavity, which may be implicated in their disrupted antigen presenting capacity. Moreover, we showed an elevated level of sGal-9 and sTIM-3 in the PF of EMS patients in comparison to the plasma. Additionally, we showed an elevated level of plasma sGal-9 in the EMS patients in comparison to the healthy subjects. Interestingly, the concentration of sGal-9 was significantly elevated in the plasma in both the late (III/IV) and early (I/II) ASRM stages of EMS in relation to the control group. Our results led us to conclude that the accumulation of Gal-9 expressing mDCs and pDCs in the PF and the high sTIM-3/sGal-9 production in the peritoneal cavity could represent the hallmark of immune regulation in EMS patients, which may augment the inflammatory process and development/maintenance of local immunosuppression. A better understanding of the immunotolerant microenvironment in the peritoneal cavity and the interactions between the lesions and immune cells in EMS patients will provide the background for discovering innovative therapeutic methods. Therefore, other functional studies should be conducted to prove the role of ICPs in EMS.

## Figures and Tables

**Table 1:** Percentage of Gal-9-positive dendritic cells in patients with endometriosis and the control group.

Peripheral Blood EMS		
%	Median	Range
BDCA-1^+^CD19-Gal-9^+^ total (*n* = 77)	6.61	0.20–40.98
BDCA-1^+^CD19-Gal-9^+^ I/II ASRM stages (*n* = 34)	4.09	0.69–26.55
BDCA-1^+^CD19-Gal-9^+^ III/IV ASRM stages (*n* = 43)	8.31	0.20–40.98
BDCA-2^+^CD123-Gal-9^+^ total (*n* = 82)	2.95	0.41–44.58
BDCA-2^+^CD123-Gal-9^+^ I/II ASRM stages (*n* = 38)	2.57	0.41–44.58
BDCA-2^+^CD123-Gal-9^+^ III/IV ASRM stages (*n* = 44)	3.33	0.46–36.43
Peritoneal Fluid EMS		
%	Median	Range
BDCA-1^+^CD19-Gal-9^+^ total (*n* = 20)	45.30	2.25–85.29
BDCA-1^+^CD19-Gal-9^+^ I/II ASRM stages (*n* = 13)	44.57	4.13–85.29
BDCA-1^+^CD19-Gal-9^+^ III/IV ASRM stages (*n* = 7)	46.02	2.25–83.91
BDCA-2^+^CD123-Gal-9^+^ total (*n* = 23)	44.44	0.05–86.84
BDCA-2^+^CD123-Gal-9^+^ I/II ASRM stages (*n* = 15)	28.47	0.05–86.84
BDCA-2^+^CD123-Gal-9^+^ III/IV ASRM stages (*n* = 8)	53.41	2.25–75.23
Peripheral Blood Control Group		
%	Median	Range
BDCA-1^+^CD19-Gal-9^+^ (*n* = 10)	2.67	1.11–10.81
BDCA-2^+^CD123-Gal-9^+^ (*n* = 10)	2.09	0.97–10.07

**Table 2:** Levels of sGal-9 (ng/mL) and sTIM-3 (pg/mL) in the plasma and PF of EMS patients and in the plasma of healthy donors.

Endometriosis					
	Peripheral Blood			Peritoneal Fluid	
	Median	Range		Median	Range
sGal-9 (ng/mL) (*n* = 77)	6.75	4.21–14.82	sGal-9 (ng/mL) (*n* = 35)	11.65	5.31–32.23
sTIM-3 (pg/mL) (*n* = 38)	1547.57	744.49–2297.15	sTIM-3 (pg/mL) (*n* = 20)	4569.18	3267.36–10,348.85
Peripheral Blood Control Group					
	Median			Range	
sGal-9 (ng/mL) (*n* = 19)	1.87			1.14–11.61	
sTIM-3 (pg/mL) (*n* = 19)	1290.2			460.62–2922.96	

**Table 3:** Clinical characteristics of the EMS patients group.

The Clinical Features	Patients with Endometriosis (*n* = 82)
Age (median), years (range)	32 (19–44)
Stages of EMS (the ASRM Classification System)	
Early I/II (*n* = 38)	
Stage I (minimal)	20
Stage II (mild)	18
Advanced III/IV (*n* = 44)	
Stage III (moderate)	34
Stage IV (severe)	10
Healthy subjects (*n* = 10)	
Age (median), years (range)	29 (19–44)

## Data Availability

All data generated and analyzed during this study are included in this publication.

## Supplementary Materials

The following supporting information can be downloaded at: https://www.mdpi.com/article/10.3390/ijms24065948/s1.
