# Supplementary material for: Expression of Gal-9 on Dendritic Cells and Soluble Forms of TIM-3/Gal-9 in Patients Suffering from Endometriosis
Source: Int J Mol Sci. 2023 Mar 21;24(6):5948. doi: 10.3390/ijms24065948 (PMC10056739; doi:10.3390/ijms24065948)
Supplement: Supplementary file 1 [file ijms-24-05948-s001.zip › ijms-2230137-supplementary.pdf]

Supplementary Materials

Expression of Gal-9 on dendritic cells and soluble forms of TIM-3/Gal-9 in patients suffering from endometriosis

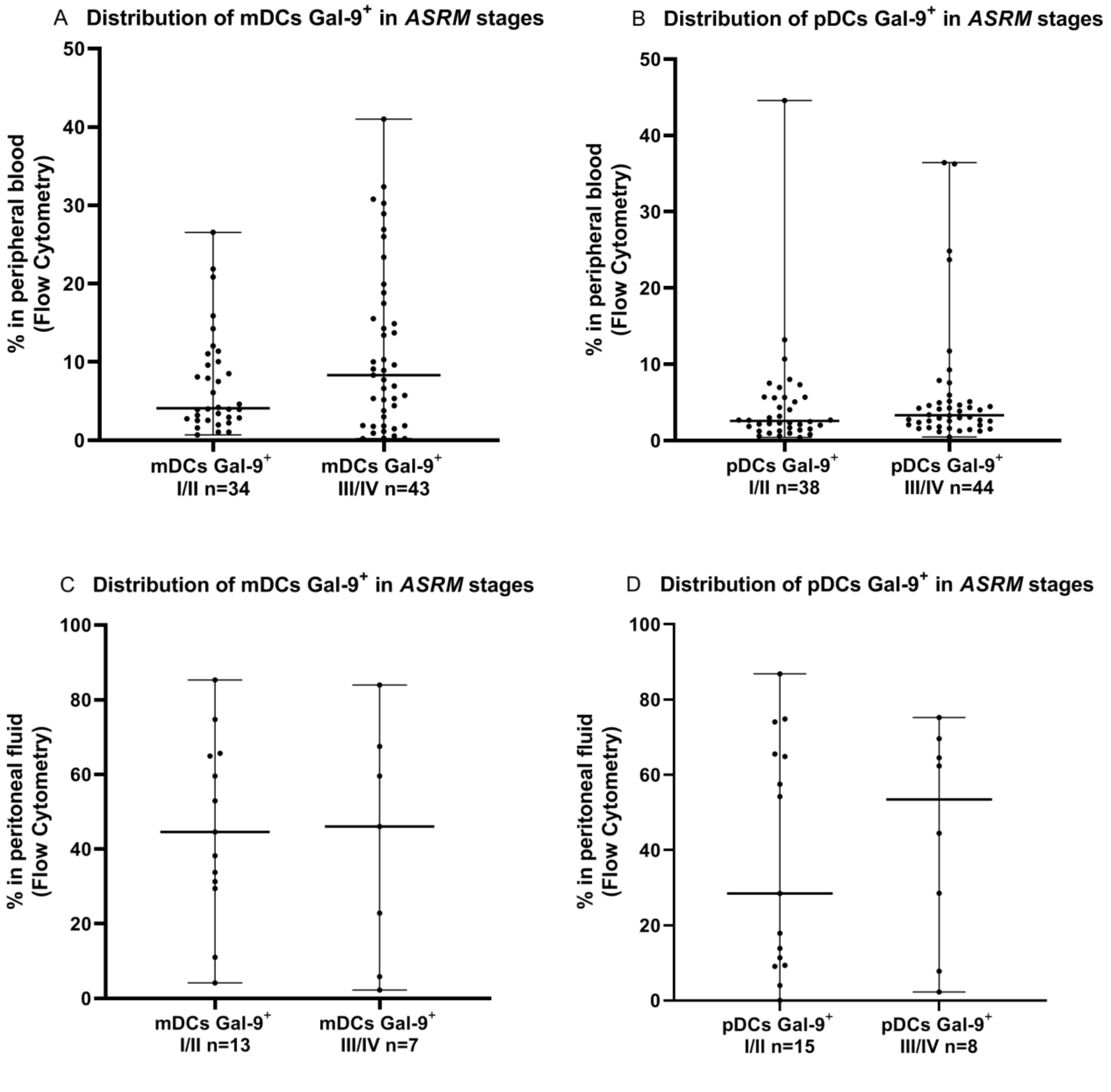

Figure S1. Percentage of mDCs and pDCs with Gal-9 expression in peripheral blood and peritoneal fluid in early (I/II) and late (III/IV) ASRM stages of endometriosis.

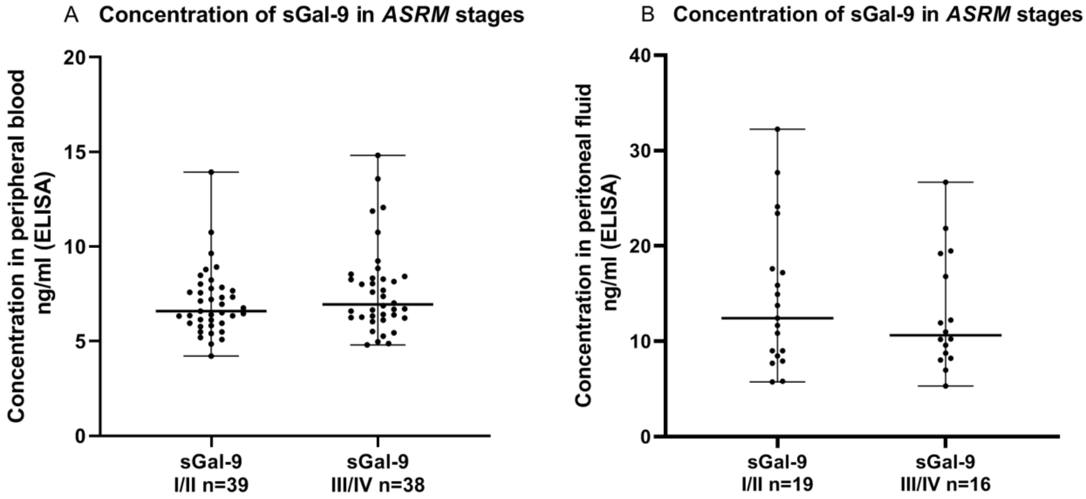

Figure S2. Level of sGal-9 in the plasma (**A**) and peritoneal fluid (**B**) in early (I/II) and late (III/IV) ASRM stages of endometriosis (ng/ml).

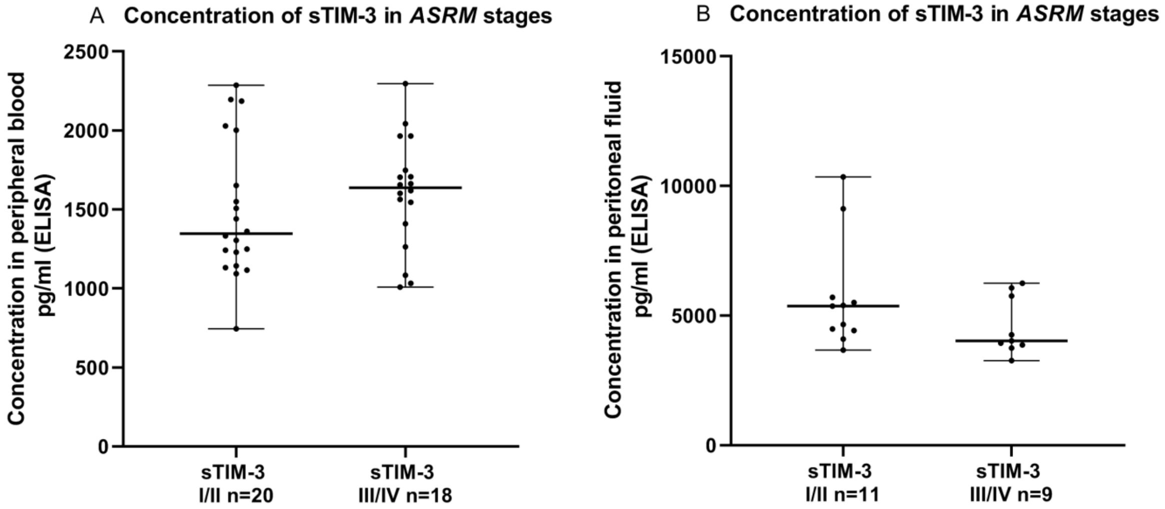

Figure S3. Level of sTIM-3 in the plasma and peritoneal fluid in early (I/II) (**A**) and late (III/IV) (**B**) ASRM stages of endometriosis (pg/ml).
